# 185. Implementation of Multiplex Polymerase Chain Reaction in Clinical Practice: Impact on Antimicrobial Management In Infectious Diarrhea

**DOI:** 10.1093/ofid/ofac492.263

**Published:** 2022-12-15

**Authors:** Gillean Kelly, Roumen Iordanov, Alex Franklin, Amna Ahmed, Krithia Srinivasan, Jesica Hayon, Todd M Lasco, Rosie Amini, Sabra Shay, Prathit A Kulkarni, Mayar Al Mohajer

**Affiliations:** Baylor College of Medicine / The University of Texas Health Science Center at Houston School of Public Health, Houston, Texas; Baylor College of Medicine, Houston, Texas; Baylor College of Medicine, Houston, Texas; Baylor College of Medicine, Houston, Texas; Stanford, Palo Alto, California; Baylor College of Medicine, Houston, Texas; Baylor St. Luke's Medical Center, Houston, Texas; Premier Healthcare Inc., Seattle, Washington; Premier, Charlotte, North Carolina; Michael E. DeBakey VA Medical Center / Baylor College of Medicine, Houston, Texas; Baylor College of Medicine, Houston, Texas

## Abstract

**Background:**

Stool culture and stool polymerase chain reaction (PCR) panels are both used to evaluate patients with suspected infectious diarrhea. Stool PCR panels are especially advantageous because of their ability to detect a broad array of infectious pathogens in less than one hour. Our study assessed how the use of stool PCR panels instead of stool culture impacted antibiotic days of therapy (DOT) and length of therapy (LOT) in hospitalized patients with suspected infectious diarrhea.

**Methods:**

In December 2021, an intervention was implemented in our hospital in which all electronic orders for stool cultures were automatically switched to stool PCR testing. The pre-intervention group was comprised of 75 hospitalized adult patients who had a stool culture obtained from September to November 2021. The post-intervention group was comprised of 81 adult patients who had a stool PCR obtained from December 2021 to February 2022. The DOT and LOT for antibiotics prescribed specifically for infectious diarrhea were determined for each patient; DOT and LOT were compared between the pre- and post- intervention groups.

**Results:**

The median DOT in the pre- and post-intervention groups was 0 with a range of 0-10 and 0-8, respectively. The median LOT in the pre- and post-intervention groups was 0 (range 0-5 for both groups). No significant difference in the median DOT (Wilcoxon rank sum test, *p*-value = 0.967) or LOT (Wilcoxon rank sum test, *p*-value = 0.993) was found between the pre- and post-intervention groups (Figure 1). After adjusting for patient days present, no significant change in DOT or LOT incidence rate was found between the pre- and post-intervention groups. The DOT incidence rate ratio (RR) was 0.71 (95% CI 0.42, 1.22), and the LOT incidence RR was 0.67 (95% CI 0.36, 1.24).

**Figure 1.**
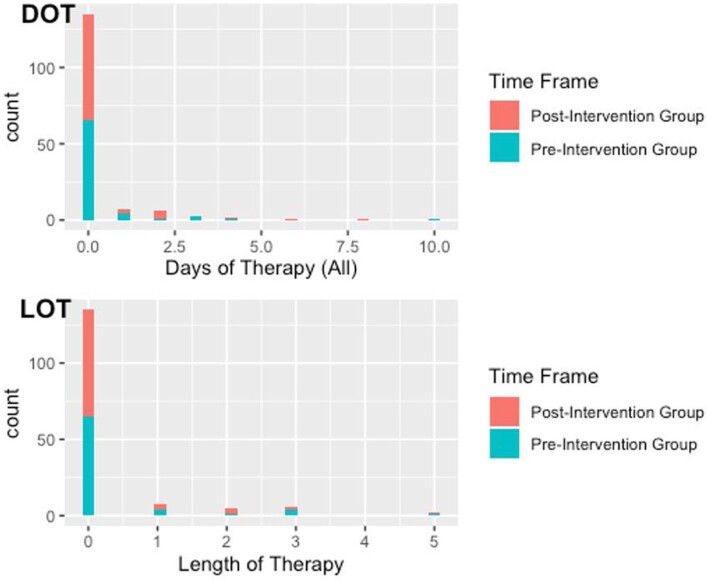
DOT and LOT Comparisons between Pre- and Post-Intervention Groups

Antibiotic days of therapy (DOT) stratified by study period (top) and antibiotic (LOT) stratified by study period (bottom).

**Conclusion:**

An intervention of automatically changing stool culture testing to stool PCR testing did not result in a significant change in median DOT or LOT in hospitalized adult patients, nor did it result in a significant change in DOT or LOT incidence rate. These findings could be explained by an insufficient sample size (n = 156), limiting the study’s power. Additionally, most patients in the pre-intervention group received no antibiotics for infectious diarrhea, resulting in a short DOT and LOT at baseline.

**Disclosures:**

**Sabra Shay, BSN, MPH**, Premier Inc.: Employee **Prathit A. Kulkarni, M.D.**, Vessel Health, Inc.: Grant/Research Support.

